# Case Report: Opioid Use Disorder Associated With Low/Moderate Dose of Loperamide in an Intellectual Disability Patient With CYP3A and P-Glycoprotein Reduced Activity

**DOI:** 10.3389/fpsyt.2022.910684

**Published:** 2022-06-23

**Authors:** Vincent Guinchat, Nicolas Ansermot, Kuntheavy Ing Lorenzini, Dimitri Politis, Youssef Daali, Chin B. Eap, Séverine Crettol

**Affiliations:** ^1^Psychiatric Section of Mental Development, Department of Psychiatry, Lausanne University Hospital, University of Lausanne, Lausanne, Switzerland; ^2^Unit of Pharmacogenetics and Clinical Psychopharmacology, Department of Psychiatry, Lausanne University Hospital, University of Lausanne, Lausanne, Switzerland; ^3^Division of Clinical Pharmacology and Toxicology, Geneva University Hospitals, Geneva, Switzerland; ^4^Addiction Medicine, Department of Psychiatry, Lausanne University Hospital, University of Lausanne, Lausanne, Switzerland; ^5^School of Pharmaceutical Sciences, University of Lausanne, University of Geneva, Geneva, Switzerland; ^6^Center for Research and Innovation in Clinical Pharmaceutical Sciences, University of Lausanne, Lausanne, Switzerland; ^7^Institute of Pharmaceutical Sciences of Western Switzerland, University of Lausanne, University of Geneva, Geneva, Switzerland

**Keywords:** loperamide, opioid use disorder, intellectual disability, cytochrome P450, P-glycoprotein, case report

## Abstract

Loperamide is an over-the-counter antidiarrheal for which increasing cases of abuse or misuse are described. We report the onset of opioid use disorder associated with low to moderate doses of loperamide in an intellectual disability patient without previous history of substance use disorder (SUD). Our patient presented strongly reduced activities of CYP3A and P-glycoprotein, which are mainly involved in loperamide metabolism and transport. We suggest that this led to an increase in bioavailability, systemic exposure, and brain penetration thus allowing loperamide to act on the central nervous system and contributing to the development of SUD. Slow release oral morphine (SROM) was chosen as opioid agonist treatment, which successfully contained loperamide use and globally improved her clinical condition. This situation highlights the need for caution and awareness when prescribing loperamide, particularly in vulnerable patients with few cognitive resources to understand the risks of self-medication and little insight into its effects.

## Introduction

Loperamide is an over-the-counter antidiarrheal medication, perceived as harmless and widely available, but cases of abuse and misuse are increasingly reported, mostly in patients with previous history of substance use disorder (SUD) (1–3). Loperamide is a potent mu-opioid receptor agonist with predominantly peripheral activity, which inhibits intestinal secretion and peristalsis (4). It has a very low systemic bioavailability due to an important first-pass metabolism, with cytochrome P450 (CYP) 3A and CYP2C8 mainly involved (4). It is also a substrate of P-glycoprotein (PGP), an ATP-binding efflux transporter, which, in addition to contributing to the low systemic bioavailability, results in poor central nervous system (CNS) penetration. Thus, the low oral absorption and the inability to cross the blood–brain barrier explains why loperamide therapy at usual dosage (≤16 mg/d) is associated with very little CNS effects (4). However, at higher doses, it has been associated with CNS effects such as euphoria, CNS depression, and even death (5). Loperamide is primarily misused as a remedy for opioid withdrawal, but is also abused as a substitute for opioids at higher doses (70–400 mg/d) (1). Loperamide ingestion has also been reported in association with PGP and/or CYP inhibitors to facilitate CNS opioid effects and advice on how best to combine them is available on several illicit drug information websites (1, 5). High doses of loperamide have been associated with cardiotoxicity issues, including QTc prolongation, torsades de pointes, and cardiac arrest (5), leading the FDA to warn of its cardiotoxicity in higher doses and to develop strategies to reduce its use (2). However, little attention is given to the fact that addiction to such drug can develop insidiously, in particular in vulnerable patients with few cognitive resources and little insight.

To our knowledge, we present here the first case of dependence on low to moderate doses of loperamide in an intellectual disability patient without previous history of substance use disorder (SUD) but presenting reduced CYP3A and PGP activity. The authors obtained written consent from the patient and her legal representative to publish this case report as well as for the use of personal data for research purposes and for the genetic analysis.

## Case Description

Mrs. A, 39 was born in the Lausanne area. She is the third child of her Italian and Portuguese parents. In early childhood, she suffered a neonatal anoxia and a pediatric hospitalization for anorexia at 2 years old. At 4, a global delay in development without socialization disorders worried her parents and pediatrician. A psychiatrist performed an assessment when she was 7 years old, which led to a diagnosis of moderate intellectual disability. Probably because she had no medical comorbidity, Mrs. A was never referred to a neuropediatrician or geneticist. Mrs. A subsequently had a long history of psychiatric follow-up for challenging behaviors such as aggression, self-injurious behaviors (self-burn), and other maladaptive behaviors. Irritability, impulsivity, somatizations, intolerance to frustration, emotional lability, and conflicts with peers are recurrent clinical features reported through her development. Most symptoms were initially assigned to a lack of coping strategies in a conflictual familial environment. At 11, she was addressed to an internship specialized institution where she followed an intense speech therapy, occupational therapy and psychotherapy. It enabled a slight improvement in her global motricity. As a teenager, she suffered two sexual assaults and was hospitalized two times in psychiatry for aggression and self-injurious behaviors. At 20, her last cognitive assessment, using a Brunet Lezine test, estimated her IQ at 40. At this time she showed abilities for learning concrete tasks (such as to travel alone, buy groceries, participate in workshops) but was unable to read or write. Despite, some similarities with the symptoms of borderline disorder diagnosis she never fulfilled all its criteria. Some neuroleptics medications such as olanzapine and risperidone had been tried temporarily to relieve her irritability. Apart from recurrent episodes of fractures, sprains, or burns (not always deliberate), she has no other medical history but used to consult frequently for somatic complaints.

At 26, as she regularly ran away from her institution, a return home was decided. The conflictual familial context still affected her but her clinical course was positive until she was 33 years old. Her parents reported then an incipient degradation of her condition following the loss of loved ones. Self-injurious behavior (repeated burns of the wrist in the oven), recurrent suicidal ideation, and somatizations were the mains features, but no medication was accepted by the patient. Retrospectively, we consider that she fulfilled criteria of depression and anxiety. Loperamide consumption started insidiously, a year later, just after a shameful diarrhea incident, in a plane, that led to the first PRN prescription. At the age of 35, her loperamide consumption became daily, and getting access to medication led to conflictual situations. When she could not take loperamide (due to occlusive symptoms or lack of opportunity to buy pills), she presented opiate withdrawal symptoms such as irritability, mydriasis, and nasal discharge, further leading to dependence on nasal decongestant spray (xylometazoline 10 ml/day). At the age of 37, she was excluded from an occupational daycare institution (due to multiple conflicts with her colleagues) and was hospitalized in psychiatry after assaulting her mother with a knife for money to buy loperamide. Her average consumption of loperamide had increased to 40 mg/d. At discharge, she was referred to our intellectual disability psychiatric unit for the management of her depression and anxiety. She then reported diarrhea as side effect of several antidepressants (venlafaxine and escitalopram) that led to poor compliance, resumption of loperamide consumption and progressive increase to 60 mg/d. After admission to the emergency ward for a sub-occlusion, she was finally diagnosed with SUD meeting 9 out of 11 DSM5 criteria. No other toxic or alcohol consumption had been reported but no routine toxicological examination was offered. No electrocardiogram (ECG) was performed while she was using loperamide, but a QTc of 444 ms was measured during the following hospitalization. The patient's main diagnosis, events, symptoms, and prescription are described in Table 1.

**Table 1:** Timeline of patient's main diagnosis, events, symptoms and prescription.

**Age**	**Diagnosis**	**Events**	**Symptoms**	**Prescription and drug use**
From 7	Moderate intellectual disability		Challenging behaviors such as aggression, self-injurious behaviors (self-burn), and other maladaptive behaviors	
33		Insipient degradation since loss of loved ones	Aggression, self-injurious behaviors (self-burn), and other maladaptive behaviors	
34		Shameful diarrhea incident		First loperamide PRN prescription
35				Loperamide daily^*^
37		Assaulting her mother with a knife for money to buy loperamide	Opiate withdrawal symptoms when loperamide not available	Loperamide 40 mg/d^*^
	Anxiety and depression	Referral to intellectual disability psychiatric outpatient unit	Diarrhea as side effect of several antidepressants	Loperamide 60 mg/d^*^ Venlafaxine, citalopram (<1 week of prescription, poor adherence) Clorazepate 10 mg/d
	Substance use disorder	Hospitalization for sub-occlusion		SROM 30 mg/d increased to 90 mg/d
39		Global clinical improvement	Only one episode of self-burn in 6 months	SROM 90 mg/d

* *non-prescription use. SROM, slow release oral morphine*.

## Intervention, Outcome and Follow-Up

The initial prescription of 30 mg/d of slow-release oral morphine (SROM) immediately led to a complete cessation of loperamide consumption, but as Mrs. A. still experienced irritability, anxiety, and loperamide cravings, SROM was progressively increased to 90 mg/d after 2 months. Global clinical improvement followed and anxiety-related diarrhea disappeared, thus allowing educative management and integration in a new occupational institution even though self-injurious behaviors continued to occur occasionally. At the age of 39 and under SROM for 2 years, Mrs. A did not use loperamide again but the dose of morphine could not be reduced because of the recurrence of craving symptoms. Her global clinical condition has improved and only one episode of self-burn was reported in the last 6 months.

## Investigations

Following the onset of opioid use disorder associated with low to moderate doses of loperamide as compared to higher doses associated with SUD in most published reports (1), Mrs. A. drug metabolizing profile was investigated for a peculiarity using phenotyping and genotyping tests. Her CYP and PGP activities were determined with the Geneva Cocktail for CYP phenotyping as previously described (6). She received an oral cocktail capsule containing low doses of the probes bupropion (CYP2B6), flurbiprofen (CYP2C9), omeprazole (CYP2C19), dextromethorphan (CYP2D6), midazolam (CYP3A), caffeine (CYP1A2) and fexofenadine (PGP). Capillary blood samples were taken after 2 h for the CYPs and 2, 3, and 6 h for PGP. The concentrations of probes/metabolites were determined in dried blood spots using a single liquid chromatography–tandem mass spectrometry method (7). At this time, Mrs. A. was prescribed citalopram but with poor medication adherence and she was taking loperamide: both drugs are not considered CYP or PGP strong inhibitors and are therefore not expected to interfere with the phenotyping test. CYP3A4^*^22 (rs35599367), *ABCB1 1236C*>*T* (rs1128503), *2677G*>*T/A* (rs2032582) and *3435C*>*T* (rs1045642) were genotyped by real-time PCR using commercialized TaqMan® SNP Genotyping Assays according to the manufacturer's instructions (ViiA 7, ThermoFisher Scientific, Rotkreuz, Switzerland).

Her phenotyping test demonstrated a slow CYP3A activity, with a 1′-hydroxymidazolam/midazolam metabolic ratio of 0.14. This value is similar to the value of 0.22 ± 0.07 measured in 10 volunteers after CYP inhibition by voriconazole as compared to 0.57 ± 0.25 in the same volunteers without inhibitor (6). Her Geneva cocktail's phenotyping results for the other CYPs included normal metabolizing status for CYP2C9 and CYP2C19, intermediate to slow for CYP1A2 and CYP2D6 and rapid for CYP2B6. Furthermore, her PGP phenotype was categorized as reduced activity. Her measured fexofenadine area under the curve (AUC) _0−6*h*_ was 482 ng^*^h/ml, a value which is even superior to the mean value measured in 10 volunteers after PGP inhibition with quinidine (286 ± 67 ng^*^h/ml) as compared to 100 ± 48 ng^*^h/ml measured in the same volunteers without PGP inhibition (6). With regard to her genotyping results, Mrs. A does not carry the CYP3A4^*^22 allele and she is a heterozygous carrier of all three PGP single nucleotide polymorphisms (SNPs) tested (*ABCB1 1236 CT; 2677 GT; 3435 CT*).

## Discussion

Loperamide is mostly metabolized by CYP3A, which is strongly influenced by environmental factors. Therefore, phenotyping tests, such as the midazolam metabolite ratio, are the most useful tools for determination of its activity. The metabolic ratio measured for Mrs. A., who was not taking any CYP interacting medication, is associated with a slow metabolism of CYP3A. This low activity has likely increased her exposure to loperamide. In comparison, the CYP3A inhibitor itraconazole have been shown to markedly increase loperamide maximum plasma concentration (2.9-fold) and systemic exposure (3.8-fold) (2). Regarding the CYP3A4 genotyping test, Mrs. A. does not carry the CYP3A4^*^22 allele, which has been linked to a slow CYP3A4 activity but is only found in about 5–7% of Caucasians (8). So far *CYP3A4* genotypes only contribute to a minor extent to the interindividual variability of CYP3A activity, the major causes of variability being regulatory factors and drug interactions (9).

Loperamide is a substrate of PGP, a transporter involved in absorption and brain penetration of drugs. Mrs. A. PGP phenotype corresponds to a markedly reduced activity, which could be in line with her genotyping results. There have been equivocal results on the impact of *ABCB1* SNPs on the pharmacokinetics of different medications in the literature, but higher concentrations might be observed, due to decreased PGP protein expression and activity, in *3435T* carriers such as Mrs. A (10). Her reduced PGP activity, as compared to the effect of the strong PGP inhibitor quinidine, could have resulted in increased bioavailability, systemic exposure and brain penetration of loperamide (4). When quinidine was co-administered, an increased loperamide central effect (measured as pupil size reduction or respiratory depression) was observed at therapeutic doses of loperamide (2). Therefore, combined with low CYP3A activity, the central effect of loperamide associated with reduced PGP activity might have become sufficiently important to induce the development of SUD. Initially, Mrs. A. loperamide consumption was a misuse to treat phobic and anxiety disorder induced by diarrhea. We assumed that due to her reduced CYP3A and PGP activities she experienced central opioid analgesic effect, also potentially reducing her stress that subsequently led to severe opioid use disorder. The SUD diagnosis was delayed due to her lack of insight and her phobic symptoms, which diminished markedly after opioid agonist treatment prescription.

Limited data is available on treatment options for loperamide use disorder. Methadone and buprenorphine tapering are described in case reports (11–13) and two case series described long-term buprenorphine treatment (14, 15), but the utility of long-term maintenance treatment remains unknown. For Mrs. A., SROM treatment was chosen to avoid CYP metabolism and to reduce adverse events associated with loperamide, such as QTc interval prolongation. Her clinical condition has much improved under morphine treatment and she has not used loperamide again. However, until now, the tapering of morphine was not successful.

In conclusion, our patient's reduced CYP3A and PGP activities might have been risk factors to develop loperamide dependence in addition to a pre-existing psychiatric condition with few cognitive resources to understand the risks of self-medication and little insight into its effects. Mrs. A. pharmacokinetic peculiarities could have caused the development of SUD in a patient without previous history of SUD with use of low to moderate doses of loperamide as compared to doses previously described for euphoric effect (1, 2). This clinical situation highlights the need for caution and awareness when prescribing and using over-the-counter medications such as loperamide, in particular in vulnerable patients with few cognitive resources to understand the risks of self-medication and little insight into its effects.

## Data Availability Statement

The original contributions presented in the study are included in the article, further inquiries can be directed to the corresponding author.

## Ethics Statement

Written consent was obtained from the patient and her legal representative to publish this case report as well as for the use of personal data for research purposes and for genetic analysis.

## Author Contributions

VG and DP provided care to the patient. VG and SC drafted the manuscript with support from NA, KIL, YD, and CBE. All authors contributed to manuscript revision, read, and approved the submitted version.

## Funding

This research received no external funding, except for the Open access funding provided by University of Lausanne.

## Conflict of Interest

The authors declare that the research was conducted in the absence of any commercial or financial relationships that could be construed as a potential conflict of interest.

## Publisher's Note

All claims expressed in this article are solely those of the authors and do not necessarily represent those of their affiliated organizations, or those of the publisher, the editors and the reviewers. Any product that may be evaluated in this article, or claim that may be made by its manufacturer, is not guaranteed or endorsed by the publisher.
